# Evaluation of the Pathogenicity of Highly Virulent Eurasian Genotype II African Swine Fever Virus with MGF505-2R Gene Deletion in Piglets

**DOI:** 10.3390/v17121565

**Published:** 2025-11-29

**Authors:** Fan Xu, Huaguo Huang, Wen Dang, Yu Du, Tao Li, Huanan Liu, Zhengwang Shi, Hong Tian, Jijun He, Haixue Zheng

**Affiliations:** 1 State Key Laboratory for Animal Disease Control and Prevention, Lanzhou Veterinary Research Institute, Chinese Academy of Agricultural Sciences, College of Veterinary Medicine, Lanzhou University, Lanzhou 730000, China; 82101221330@caas.cn (F.X.);; 2 Ecology and Agriculture College, Sichuan Minzu College, Kangding 626001, China; 230003@scun.edu.cn

**Keywords:** African swine fever virus, MGF505-2R, live attenuated vaccine, pathogenicity, immune response

## Abstract

African swine fever virus (ASFV) poses a significant threat to the global pig industry due to high mortality rates and complex genetic variation. Live attenuated vaccines (LAVs) provide protection against ASFV. Previously, MGF505-2R was identified as a potent inhibitor of innate immunity in vitro. This study evaluates the pathogenicity of a recombinant Eurasian genotype II strain with the MGF505-2R gene deleted (ASFV-ΔMGF505-2R) in piglets. Twelve five-week-old crossbred piglets were divided into two groups, with one group of eight piglets inoculated with ASFV-ΔMGF505-2R (*n* = 8) and the other group of four piglets inoculated with the same dose of parental ASFV CN/GS 2018 (*n* = 4). Clinical symptoms, viral loads, and immune responses were monitored over 30 days. ASFV-ΔMGF505-2R-inoculated piglets exhibited transient fever and low viremia only in the beginning of the challenge, while the control group developed high levels of viremia and hyperthermia at day 2 and 8 post-challenge, respectively. Meanwhile, the control group demonstrated more severe post-mortem signs and immuno-histochemistry injury when compared to the ΔMGF505-2R group. ELISA analysis displayed higher levels of IFN-β and IL-1β in the ΔMGF505-2R group, further solidating the immunosuppressive role of MGF505-2R. All ASFV-ΔMGF505-2R-inoculated piglets developed high titers of ASFV-specific P30 antibodies at 10 days post-challenge. These findings rationalized the potential of ASFV-ΔMGF505-2R as a live attenuated candidate for ASF infection.

## 1. Introduction

African swine fever virus (ASFV) is a complex, enveloped, double-stranded DNA virus belonging to the *Asfarviridae* family and is the only known DNA arbovirus capable of being transmitted by soft ticks of the genus *Ornithodoros* in certain endemic regions [1,2]. The virus mainly infects domestic pigs and wild boars, causing a highly contagious and often fatal disease characterized by high fever, hemorrhages, lymphoid depletion, and multi-organ failure [3]. Mortality rates reached up to 100% when pigs are infected with highly virulent strains, making ASF one of the most devastating swine disease worldwide [4].

The socioeconomic impact of ASF outbreaks extends far beyond animal health, posing a severe threat to global food security. Since initial identification in Kenya in 1921, ASFV has been spreading out of Africa to Europe, Asia, and more recently America. Since its first introduction into China in 2018, large-scale outbreaks have been reported in Vietnam, India, the Philippines, and Indonesia [1,5,6,7]. The transcontinental spread of ASFV highlights the role of key prevention and control technologies, including strengthened biosecurity, animal movement control, and early detection.

Development of a safe and effective ASF vaccine remains a major challenge in the veterinary field because of its large genome, sophisticated replication processes, host immune evasion, genetic variability and unknown functions of novel proteins [8,9,10,11]. Among the various strategies for developing vaccines against ASF, live attenuated vaccines (LAVs), as commonly generated through serial passage in cell culture or targeted deletion of virulence-associated genes, hold great promise [12,13,14]. LAVs mimic natural infection without causing severe disease, thereby stimulating both humoral and cellular immune responses. Several experimental LAVs have partial to complete protection against homologous, in some cases heterologous challenges in laboratory settings. Field trials in endemic areas have also provided encouraging evidence of high efficacy in the context of AVAC ASF LIVE^®^ and NAVET-ASFVAC^®^, two ASFV LAVs licensed and commercialized in Vietnam [15,16].

In recent years, various LAV candidates have been developed by deleting single or multiple genes, including members of the multigene family (MGF), such as MGF360 and MGF505 [17], I177L [18], 9GL and UK [19]. These candidate strains have exhibited varying levels of protective efficacy in vivo. Our previous study identified MGF505-2R as a potent inhibitor of innate immunity in vitro. Very recently, deletion of the MGF505-2R gene activates the cGAS-STING pathway in vitro and leads to attenuation and protection against lethal challenge in the context of ASFV strain Arm/07/CBM/C2 (LR812933.1) [20]. However, how the deletion of MGF505-2R affects the pathogenicity of the highly pathogenic Eurasian genotype II strain is unknown. We aim to evaluate the pathogenicity of ASFV-ΔMGF505-2R in piglets and explore its potential as a live attenuated vaccine candidate.

## 2. Materials and Methods

### 2.1. Animals

Twelve five-week-old crossbred pigs weighing approximately 10 kg were obtained from a commercial pig farm. All pigs tested negative for ASFV, classical swine fever virus (CSFV), porcine circovirus type 2 (PCV2), pseudorabies virus (PRV), and porcine reproductive and respiratory syndrome virus (PRRSV) antigens and antibodies. The experiments were conducted in a Biosafety Level 3 laboratory (BSL-3) at Lanzhou Veterinary Research Institute, Chinese Academy of Agricultural Sciences (CAAS). The experiment was approved by the Committee on Animal Research and Ethics of Lanzhou Veterinary Research Institute (LVRI) (Approval Code: SYXK(甘)2020-0010).

### 2.2. Virus and Cells

The parental virus strain ASFV CN/GS 2018 was preserved in our institute. The MGF505-2R gene deletion strain, ASFV-ΔMGF505-2R, was constructed by homologous recombination as described previously [21]. Porcine bone marrow-derived macrophages (BMDMs) were obtained and maintained in RPMI 1640 medium containing 20% fetal bovine serum (FBS) with 1% penicillin-streptomycin (P/S) and 2 mM L-glutamine. Meanwhile, 10 ng/mL of recombinant porcine granulocyte-macrophage colony-stimulating factor (GM-CSF) was added into the complete culture medium to maintain optimal cell viability and physiological state of BMDMs [22].

### 2.3. Evaluation of Pathogenicity of ASFV-ΔMGF505-2R

Twelve piglets were randomly split into two groups. The group of 8 piglets received an intramuscular (IM) injection of 10^2^ HAD_50_ of ASFV-ΔMGF505-2R, while the other group of 4 piglets was administered the same dose of parental ASFV CN/GS 2018. Daily body temperature measurements were taken, and clinical manifestations were recorded during the entire experimental period. Blood and fecal swabs were gathered every other day after infection [23]. Viremia and virus shedding were assessed using quantitative PCR. At necropsy, tissue specimens including the heart, liver, spleen, lung, kidney, submandibular lymph nodes, hepatogastric lymph nodes, and mesenteric lymph nodes were harvested and prepared for histopathological examination.

### 2.4. Anesthesia Procedure

For pigs that reached the predefined human endpoint or undertook all the experiments, they were securely restrained and injected IM with an overdose of Zoletil^®^50 (Virbac, Tianjin, China) at 16 mg per kilogram of body weight to induce anesthesia. The depth of anesthesia was monitored every 5 to 10 min by assessing muscle relaxation, cessation of voluntary movement, absence of palpebral reflex, and loss of consciousness [24]. Later, animals were humanely euthanized. All procedures involving anesthesia and euthanasia were carried out in strict compliance with animal welfare regulations and by following the “Standard Operating Procedures for Anesthesia and Euthanasia of Laboratory Animals” issued by the Lanzhou Veterinary Research Institute, Chinese Academy of Agricultural Sciences.

### 2.5. Immune Response Analysis

Serum specimens were tested for the presence of p30 antibodies with an ASFV p30 blocking ELISA kit (IDvet, Qingdao, China). The concentrations of IFN-β (Solarbio, Beijing, China) and IL-1β (Solarbio, Beijing, China) were determined using commercial ELISA kits.

### 2.6. The Quantitative Real-Time Polymerase Chain Reaction (q-PCR) Assay

Genomic DNA of ASFV was isolated from BMDMs or tissue samples using the E.Z.N.A.^®^ Tissue DNA Kit (OMEGA, Norcross, GA, USA) following the manufacturer’s instructions. Quantitative PCR was performed on a QuantStudio system (Applied Biosystems, Shanghai, China) with the Pro taq HS premix probe qPCR kit (ACCURATE BIOLOGY AG, Changsha, China) to measure the copy numbers of ASFV P72 genes in cell cultures, blood, swabs, and tissues.

### 2.7. Statistical Analysis

Data are expressed as mean ± standard error of the mean (SEM). Group comparisons were conducted using the t-test in GraphPad Prism 5.0 (GraphPad Software Inc., La Jolla, CA, USA). A *p* value below 0.05 was considered statistically significant (*, *p* < 0.05; **, *p* < 0.01; ***, *p* < 0.001).

## 3. Results

### 3.1. Pathogenicity of ASFV-ΔMGF505-2R in Piglets

**Figure 1:**
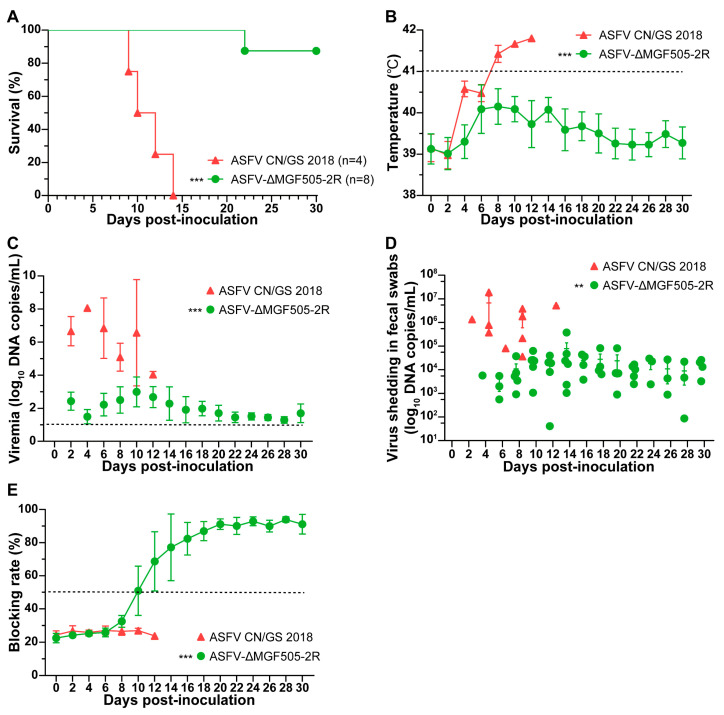
Analysis of survival rates, rectal temperatures and viral loads in the control group and ASFV-∆MGF505-2R group. (**A**) Survival rates in the two groups. (**B**) Rectal temperatures in the two groups. Analysis of viral loads using TaqMan-PCR in the (**C**) blood serum and (**D**) fecal swabs from the two groups at indicated time. (**E**) The kinetics of P30 antibody response in serum from the two groups. The dashed line represents the cut-off of blocking ELISA. The t-tests were performed for statistical analysis between the different groups (**, *p* < 0.01; ***, *p* < 0.001).

ASFV CN/GS 2018-inoculated piglets exhibited high fever (over 41.0 °C) by 6–8 days post-inoculation (dpi), followed by ASF-compatible clinical manifestations, then all died within 14 dpi (Figure 1A,B). In contrast, piglets inoculated with ASFV-ΔMGF505-2R showed transient fever only at the beginning of the challenge experiment. One pig started to show clinical signs of disease (anorexia, depression, and fever) and died at 22 dpi, while the remaining seven animals were clinically healthy throughout the entire experiment, except for the transient increase in body temperature (Figure 1A,B). Therefore, deletion of MGF505-2R led to attenuation of the ASFV CN/GS 2018 strain.

In monitoring the viremia and viral shedding in fecal swabs of both groups, we found that the control group developed high levels of viremia and more viral shedding in swabs, approximately 10^6^ to 10^8^ copies/mL by 2 dpi, remaining at peak titers until death. However, the ASFV-ΔMGF505-2R group showed a slight increase in viremia at 10 dpi and declined to almost undetectable levels. Viral shedding was significantly lower when compared to the control group, but still remained at relatively moderate concentrations ranging from 10^3^ to 10^5^ copies/mL throughout the experiment (Figure 1C,D). Therefore, ASFV-ΔMGF505-2R was highly attenuated in piglets.

To explore whether the attenuated strain could exert antibody response, we found that ASFV-ΔMGF505-2R was capable of inducing P30 antibodies even at 10 dpi. The antibody titers increased, peaked at 20 dpi and remained at plateau levels for the rest of the days. Those data support the strong immunogenicity of ASFV-ΔMGF505-2R in piglets (Figure 1E).

### 3.2. ASFV-ΔMGF505-2R Induced Slight Pathological Damage in Organs

**Figure 2:**
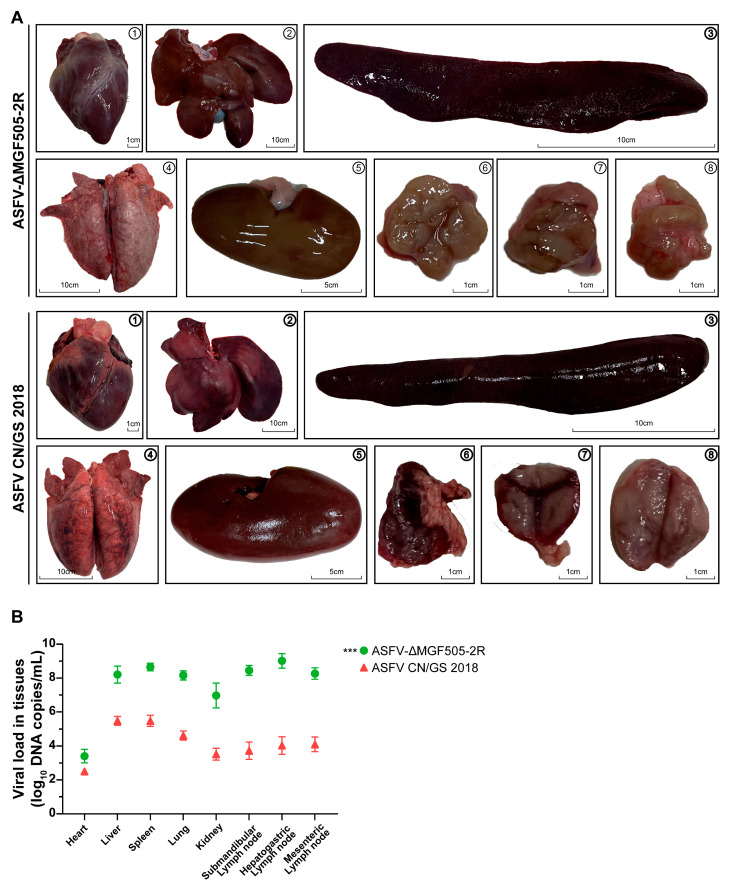
Macroscopic analysis of lesions in tissues and organs and quantification of viral loads. (**A**) Comparative postmortem lesions. The images show representative organs from pigs in each group as follows: ① heart; ② liver; ③ spleen; ④ lung; ⑤ kidney; ⑥ submandibular lymph node; ⑦ hepatogastric lymph node and ⑧ mesenteric lymph node. (**B**) Virus titers in tissues from 2 groups (red, ASFV CN/GS 2018, *n* = 4; green, ASFV-ΔMGF505-2R, *n* = 8). A total of 20 mg of tissue samples were homogenized, vortexed, clarified, and subjected to copy number detection (***, *p* < 0.001).

Organs were systematically examined for macroscopic and pathological changes, with a focus on indicators such as organ swelling, discoloration, and vascular congestion [25]. Gross examination revealed that the spleen and lungs from control group displayed a markedly enlarged size and a uniform dark red to nearly black appearance, consistent with severe splenic congestion and infarction. Widespread areas of hemorrhagic necrosis were evident across the parenchyma, indicating extensive tissue damage due to uncontrolled viral replication and associated inflammatory responses. The kidney and liver also showed pronounced abnormalities, being swollen and discolored with a dull, dark brownish-red surface, which was characteristic of renal congestion and possible microvascular thrombosis. These macroscopic findings were suggestive of impaired blood flow and potential ischemic injury in renal tissues. Additionally, the submandibular, hepatogastric and mesenteric lymph nodes were significantly enlarged and exhibited intense reddening due to marked vascular engorgement and inflammatory cell infiltration, further supporting systemic infection of the virus in control piglets (Figure 2A).

In contrast, pigs from the ASFV-ΔMGF505-2R group exhibited considerably slightly pathological manifestations. Macroscopic evaluation of their organs revealed only moderate enlargement and minimal discoloration, with no evidence of widespread hemorrhage or necrosis (Figure 2A).

Moreover, quantitative assessment of viral loads demonstrated significantly lower levels of viral DNA in the tissues of the ASFV-ΔMGF505-2R group when compared to the control group (Figure 2B). This reduction in viral replication efficiency correlates directly with the observed attenuation in clinical symptoms and organ pathology. These findings highlighted the importance of the MGF505-2R gene in viral pathogenesis and support its potential as a target for the development of a live-attenuated vaccine against ASF.

### 3.3. ASFV-ΔMGF505-2R Group Showed Only Mild to Moderate Histological Changes

**Figure 3:**
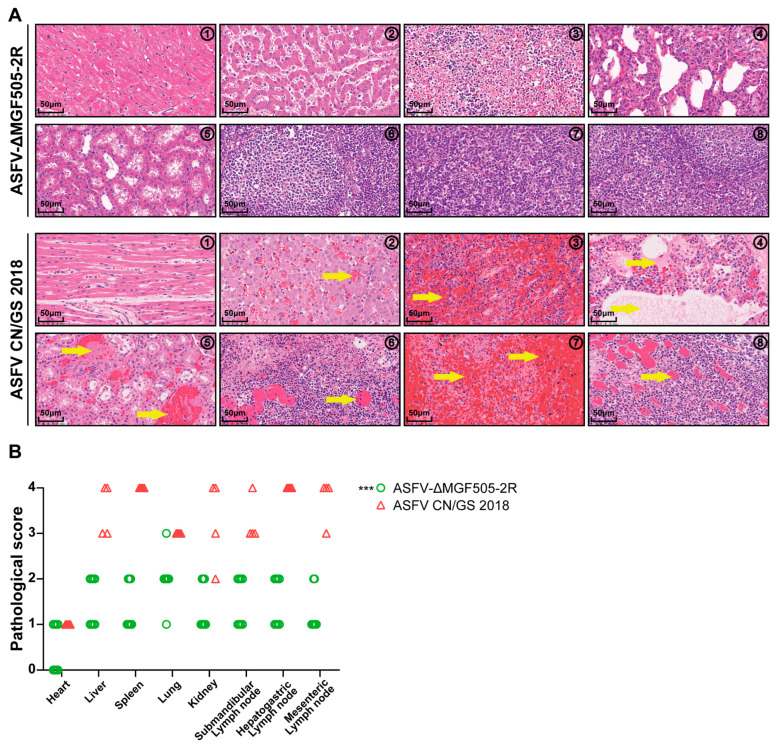
Histopathological assessment through HE staining and semi-quantitative scoring analysis of tissue damage. (**A**) Representative histopathological lesions in different tissue samples of piglets in each group. Yellow arrows indicate acute and diffuse hemorrhages. (**B**) Scores of histopathological lesions. The histopathological lesions of each pig were scored for clinical presentations of respective organs (***, *p* < 0.001).

To further characterize the extent of pathological damage, tissue samples were collected from both groups at the end of the experiment [26]. Based on the scoring criteria outlined [27], a comprehensive histopathological evaluation demonstrated that the control group exhibited severe pathological damage. The spleen exhibited extensive hemorrhage characterized by red pulp congestion and widespread necrosis of lymphocytes, consistent with the acute phase of ASFV infection. The lungs revealed marked shedding of bronchial epithelial cells, disruption of the respiratory epithelium, and the presence of serous exudates within both blood vessels and bronchioles. Furthermore, diffuse infiltration of inflammatory cells, predominantly macrophages and neutrophils, was evident within pulmonary lobules, accompanied by alveolar wall thickening and parenchymal destruction. Renal tissues displayed notable interstitial hemorrhage and focal areas of tubular epithelial cell necrosis, suggestive of impaired renal function and possible toxin-mediated or ischemic injury secondary to systemic viremia. The liver and associated lymphoid tissues also showed significant pathological damage. Specifically, three lymph nodes demonstrated disintegration of lymphoid nodules, loss of normal follicular architecture, and extensive lymphocyte depletion with nuclear fragmentation and cytoplasmic eosinophilia, the morphological hallmarks of apoptosis and necrosis (Figure 3A). These changes were indicative of systemic vascular damage and immune system collapse, which were hallmark features of highly pathogenic ASFV infections.

In stark contrast, pigs that survived the ASFV-ΔMGF505-2R strain exhibited markedly reduced tissue damage. Histopathological analysis revealed either the absence of detectable lesions or only mild, localized pathological changes in the spleen, kidney, lung, submandibular lymph node, hepatogastric lymph node and mesenteric lymph node. Notably, the structural integrity of lymphoid tissues was largely preserved, with minimal inflammatory infiltration and no evidence of widespread necrosis (Figure 3A).

Quantitative assessment of lesion scores demonstrated that the pathological damages in the ASFV-ΔMGF505-2R group were markedly slight when compared to the control group (Figure 3B). This data strongly indicates that the deletion of the MGF505-2R gene plays a critical role in attenuating ASFV. Collectively, these results rationalized that the ASFV-ΔMGF505-2R mutant strain induced mild histopathological changes, highlighting its potential as a candidate for further development in vaccine research (Supplementary Materials).

### 3.4. Immune Response Analysis

**Figure 4:**
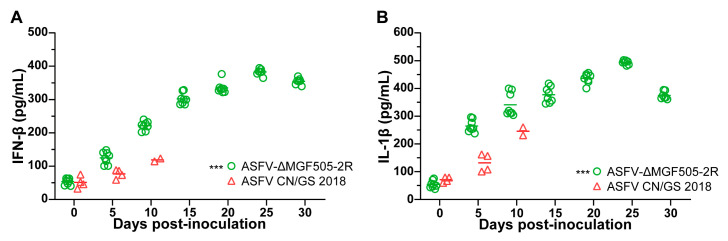
ASFV-ΔMGF505-2R inoculation induces higher IFN-β and IL-1β expression in pigs. ELISA analysis of (**A**) IFN-β and (**B**) IL-1β levels in the serum collected from piglets at the indicated time points (***, *p* < 0.001).

Viral infection can trigger a robust immune response in the host. MGF505-2R was previously identified as a potent inhibitor of innate immunity in vitro [20,21,28]. ELISA analysis revealed that pigs inoculated with ASFV-ΔMGF505-2R produced slightly higher levels of IFN-β and IL-1β compared to those in the control group, suggestive of the immunosuppressive properties of MGF505-2R in vivo (Figure 4A,B).

## 4. Discussion

### 4.1. Progress in Live Attenuated Vaccine Development

LAVs have emerged as a promising strategy for combating ASFV. Previous research has implied that targeted deletion of specific Multigene Family (MGF) genes can effectively attenuate ASFV and elicit protective immunity in pigs. For example, the deletion of MGF360 and MGF505 genes has been demonstrated to mitigate viral pathogenicity and confer protection in pigs against lethal challenge [17,29,30]. Consistent with this, the deletion of the MGF505-7R gene has also been reported to attenuate the virus and induce a protective response [31,32,33]. Sunwoo et al. have recently corroborated the pivotal role of the MGF505-2R gene in modulating the cGAS-STING signaling pathway through a series of in vitro and in vivo experiments, thereby further confirming the role of MGF505-2R in immunosuppression and viral attenuation [20]. These findings underscore the significant impact of the MGF505-2R gene on ASFV virulence and validate that its deletion markedly diminishes the pathogenic potential of the virus. Collectively, our research further substantiates the potential utility of MGF gene deletions in the development of LAVs for ASFV.

### 4.2. ASFV-ΔMGF505-2R Was Largely Attenuated in Piglets

Regarding the immune response, Sunwoo et al. elucidated the molecular mechanism by which the MGF505-2R protein inhibited the cGAS-STING signaling pathway, thereby modulating the production of IFN-β via in vitro experimental approaches, which was consistent with our findings [20]. In this study, deletion of MGF505-2R in ASFV enhanced the immune response in pigs, characterized by elevated levels of IFN-β and IL-1β, further supporting the potential of ASFV-ΔMGF505-2R as an inhibitor of innate immunity. These findings indicate that the deletion of the MGF505-2R gene not only weakens the virulence of the virus but also enhances the host immune response, thereby contributing to a more comprehensive understanding of ASFV MGF gene function.

### 4.3. Correlation Between Antibody Levels and Survival Rates

The elevated antibody levels and high survival rate observed in the ASFV-ΔMGF505-2R group indicate a positive correlation between antibody production and protection against ASFV, aligning with prior research that LAVs elicit robust immune responses and protect pigs from lethal ASFV challenge. Sunwoo et al. previously confirmed the partial protective efficacy of ASFV-ΔMGF505-2R through animal challenge experiments with protection rates of up to 75%, which needs further optimization to enhance protection [20].

Our study reinforces the evidence that targeted deletion of MGF genes can facilitate the development of effective LAVs for ASFV. The recombinant virus lacking the MGF505-2R gene emerges as a promising candidate for a modified live vaccine. This investigation underscores that the recombinant virus was significantly attenuated in animals and induced a potent immune response, thereby providing a theoretical foundation for the development of modified live vaccines.

### 4.4. Limitations of the Study and Future Perspectives

This study demonstrates the attenuation and activation of innate immunity induced by the ASFV-ΔMGF505-2R gene-deleted strain in pigs, thereby providing preliminary evidence for the role of MGF505-2R in virulence regulation and immune modulation. However, several limitations warrant consideration. First, a challenge study to directly evaluate protective efficacy was not conducted. Therefore, it remains unclear whether this strain can confer protection against homologous or heterologous ASFV strains. While activation of innate immunity, such as enhanced IFN-β secretion, is a critical prerequisite for vaccine development, it is not sufficient to ensure protective immunity. For instance, ASFV infection may elicit robust innate immune responses, yet fail to clear the virus in the absence of neutralizing antibodies or antigen-specific T-cell responses [34]. Second, although the attenuation of ASFV-ΔMGF505-2R may result from impaired viral replication, this does not necessarily indicate the induction of effective adaptive immunity. Notably, the naturally attenuated strain OURT88/3 can persist in pigs without conferring full protection upon challenge [35]. Furthermore, existing evidence indicates that the protective efficacy of live-attenuated ASFV vaccines is influenced by viral genetic homology. For example, ASFV-G-ΔMGF and ASFV-G-ΔI177L confer 100% protection against genotype II strains but show limited efficacy against emerging genotype I/II recombinant strains [16]. The current study did not define the breadth of protection conferred by ASFV-ΔMGF505-2R, which limits its potential applicability. Future studies should include comprehensive challenge experiments using varied doses and administration routes, particularly the oronasal route to better mimic natural infection. Cross-protection should be evaluated using diverse viral genotypes, including genotype I, genotype II, and recombinant strains. In addition to standard monitoring parameters (e.g., body temperature, viral shedding in swabs, viremia, and neutralizing antibody titers), detailed immunophenotyping via flow cytometry should be performed to assess CD4^+^ and CD8^+^ T cell dynamics and functional profile, thereby elucidating the mechanisms underlying protective immunity. This study did not perform homologous challenge experiments using a parental strain, nor was the protective efficacy of the ASFV-ΔMGF505-2R strain evaluated. Therefore, the potential of ASFV-ΔMGF505-2R as a vaccine candidate remains to be confirmed through future challenge studies. Such data will be essential to substantiate the potential of ASFV-ΔMGF505-2R as a viable candidate vaccine.

## 5. Conclusions

The deletion of the MGF505-2R gene in ASFV significantly reduces its virulence and enhances the host immune response in piglets. The recombinant virus ASFV-ΔMGF505-2R shows promise as a live attenuated vaccine candidate. Future studies should focus on the characterization of immune mechanisms involved and evaluating the long-term efficacy and safety of ASFV-ΔMGF505-2R in larger cohorts and under field conditions.

## Data Availability

The original contributions presented in this study are included in the article. Further inquiries can be directed to the corresponding authors.

## Supplementary Materials

The following supporting information can be downloaded at: https://www.mdpi.com/article/10.3390/v17121565/s1, Table S1: Pathological scoring criteria (health (0), minimal (1), mild (2), moderate (3), severe (4), critical (5)).

## Abbreviations

The following abbreviations are used in this manuscript:

ASFV	African swine fever virus
LAVs	Live attenuated vaccines
MGF	multigene family
dpi	days post-inoculation
