# Supplementary material for: Evaluation of the Pathogenicity of Highly Virulent Eurasian Genotype II African Swine Fever Virus with MGF505-2R Gene Deletion in Piglets
Source: Viruses. 2025 Nov 29;17(12):1565. doi: 10.3390/v17121565 (PMC12737702; doi:10.3390/v17121565)
Supplement: Supplementary file 1 [file viruses-17-01565-s001.zip › viruses-3986093-supplementary.pdf]

Evaluation of the Pathogenicity of Highly Virulent Eurasian Genotype Ⅱ African Swine Fever Virus with MGF505-2R Gene Deletion in Piglets

Supplemental Flies

Table S1 Pathological scoring criteria (health (0), minimal (1), mild (2), moderate (3), severe (4), critical (5)).

**Organic tissue**	**Heart**					
**Lesion score**	0	1	2	3	4	5
**Lesion description**	Health	There was mild proliferation of fibrous tissue in the interstitial space of local muscle bundles in the heart tissue, mild expansion of muscle bundles with diffuse infiltration of inflammatory cells (black arrow)				
**Representative pathological presentation**	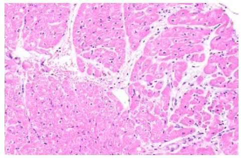	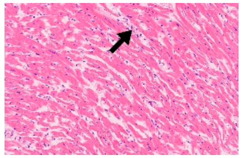				

**Organic tissue**	**Liver**					
**Lesion score**	0	1	2	3	4	5
**Lesion description**	Health	A small amount of inflammatory cells with diffuse infiltration can be seen locally in the liver tissue (black arrow)	A small amount of small focal infiltration of inflammatory cells can be seen locally in the liver tissue (black arrow); Some liver sinusoids were slightly congested, and a large number of red blood cells could be seen (green arrow)	Diffuse infiltration of inflammatory cells can be seen in some liver sinusoids of liver tissue (black arrow); Fibrous tissue hyperplasia and a large amount of collagen fiber deposition can be seen locally (blue arrow)	Focal infiltration of a large number of inflammatory cells can be seen locally in the liver tissue (black arrow); The liver sinusoids in the tissue are congested, and a large number of red blood cells can be seen gathering (green arrow); Fibrous tissue hyperplasia and a large amount of collagen fiber deposition can be seen locally (blue arrow)	Focal infiltration of a large number of inflammatory cells can be seen in the liver tissue; The liver sinusoids in the tissue are congested severely, and a large number of red blood cells can be seen (green arrow)
**Representative pathological presentation**	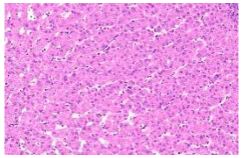	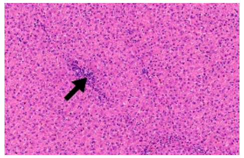	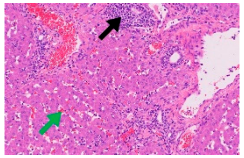	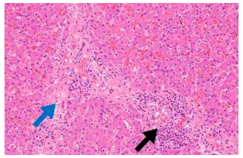	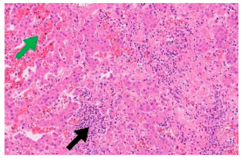	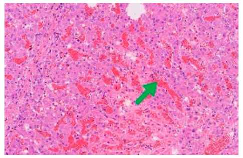

**Organic tissue**	**Spleen**					
**Lesion score**	0	1	2	3	4	5
**Lesion description**	Health	The shape of spleen nodules in spleen tissue was slightly scattered, and the boundary between red and white pulp of spleen was blurred (black arrow)	A large number of splenic nodules in spleen tissue were slightly scattered, and the boundary between red and white pulp of spleen was blurred (black arrow)	A large number of splenic nodules in spleen tissue were slightly scattered, and the boundary between red and white pulp of spleen was blurred (black arrow); The morphology of some cells disappeared; Red blood cell aggregation is visible (green arrow)	The cells in a large number of splenic nodules in spleen tissue were severely necrotic, and the morphological structure of splenic nodules was scattered, and the boundary between splenic nodules and red pulp was blurred (black arrow); Massive red blood cell aggregation can be seen in the red pulp hemorrhage of spleen tissue (green arrow)	he splenic tissue was necrotic in large area, and the structure of splenic nodules basically disappeared (black arrow); Massive red blood cell aggregation can be seen in the red pulp hemorrhage of spleen tissue (green arrow)
**Representative pathological presentation**	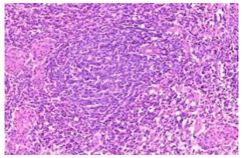	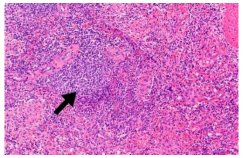	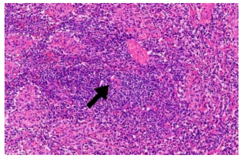	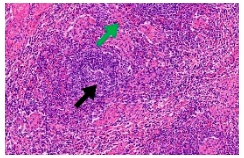	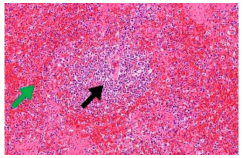	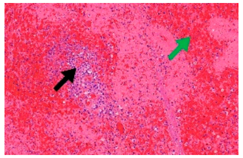

**Organic tissue**	**Lung**					
**Lesion score**	0	1	2	3	4	5
**Lesion description**	Health	Diffuse infiltration of a small number of inflammatory cells can be seen locally in the lung tissue (black arrow); Some alveolar epithelial cells in the lung tissue slightly proliferate, and the alveolar septum is thickened (blue arrow)	Diffuse infiltration of a small number of inflammatory cells can be seen locally in the lung tissue (black arrow); Some alveolar epithelial cells in the lung tissue were mildly proliferated, the alveolar septum was thickened, and a large number of alveolar cavities were atrophic (blue arrows)	Diffuse infiltration of a large number of inflammatory cells can be seen locally in the lung tissue (black arrow); Some alveolar epithelial cells in the lung tissue were mildly proliferated, the alveolar septum was thickened, a large number of alveolar cavities were atrophic, and the tissue was mildly parenchymated locally (blue arrow)	Diffuse infiltration of a large number of inflammatory cells can be seen locally in the lung tissue (black arrow); Some alveolar epithelial cells in the lung tissue were mildly proliferated, the alveolar septum was thickened, and a large number of alveolar cavities were atrophic (blue arrows); A large amount of protein mucus exudation can be seen in some alveolar cavities (yellow arrow)	Large area of cell necrosis in lung tissue, collagen deposition in tissue, massive atrophy and disappearance of alveolar cavity, severe parenchyma of tissue, accompanied by a large number of inflammatory cell infiltration (black arrow)
**Representative pathological presentation**	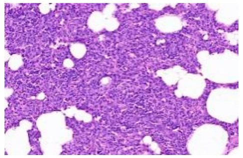	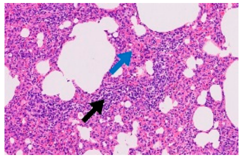	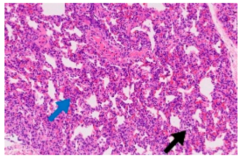	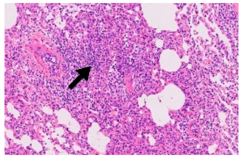	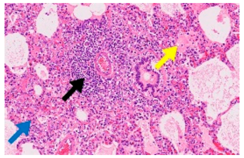	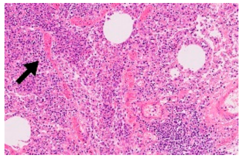

**Organic tissue**	**Kidney**					
**Lesion score**	0	1	2	3	4	5
**Lesion description**	Health	Some renal tubules were injured, and the brush border of epithelial cells fell off (blue arrow)	Some renal tubular epithelial cells in the cortical area of renal tissue were slightly edematous, with swollen cells, pale staining of cytoplasm, and irregular vacuoles (black arrows)	A large number of renal tubular epithelial cells in the cortical area of renal tissue were mildly edematous, with swelling of cells, pale staining of cytoplasm, and irregular vacuoles in the cells (black arrows); Some glomerular capsule cavities in the cortical area are dilated, and proteinaceous mucus exudation can be seen in the capsule cavity (red arrow)	A large number of renal tubules in the renal tissue were severely necrotic, and a large number of tubular epithelial cells were pyknotic (yellow arrow); Tissue tubulointerstitial blood vessels are heavily congested (green arrow)	Large area of renal tissue necrosis, a large number of tubular epithelial cells necrosis, pyknosis (yellow arrow)
**Representative pathological presentation**	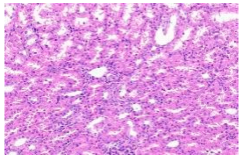	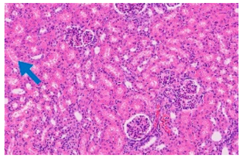	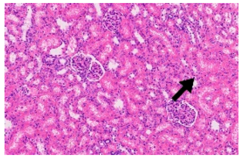	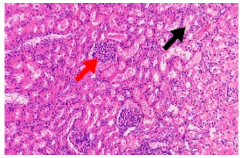	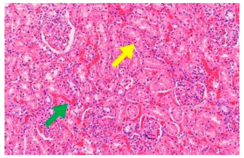	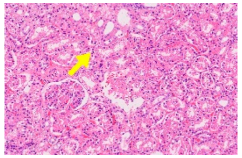

**Organic tissue**	**Submandibular Lymph Nodes**					
**Lesion score**	0	1	2	3	4	5
**Lesion description**	Health	A large number of cells in the germinal center of lymph nodes were slightly edematous, swollen and lightly stained, and the arrangement of cells in the germinal center was slightly loose and disordered (black arrow)	Some cells in the germinal center of lymph nodes were mildly edematous, with swollen cells and pale staining of cytoplasm, and the arrangement of cells in the germinal center was slightly loose and disordered (black arrow); Diffuse infiltration of inflammatory cells can be seen in the interstitial area of germinal center of tissue lymph nodes (red arrow)	Some cells in the germinal center of lymph nodes were moderately edematous, with swelling of cells and pale staining of cytoplasm, and the arrangement of cells in the germinal center was slightly loose and disordered (black arrow); Focal infiltration of inflammatory cells can be seen in the interstitial area of germinal center of tissue lymph nodes (red arrow)	Coagulative necrosis and structural destruction of some cells (green arrow)	A large number of germinal center cells in the tissue were coagulated and necrotic, the germinal center structure was severely damaged, and the boundary disappeared (green arrow)
**Representative pathological presentation**	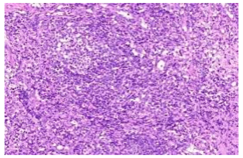	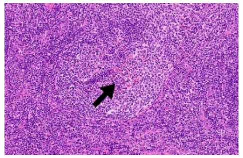	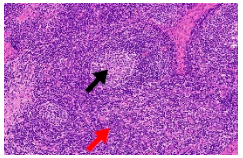	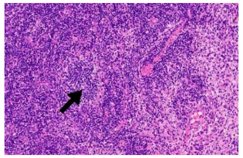	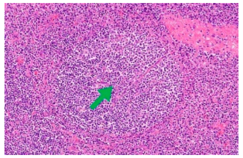	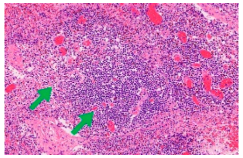

**Organic tissue**	**Hepatogastric Lymph Nodes**					
**Lesion score**	0	1	2	3	4	5
**Lesion description**	Health	Some cells in the germinal center of lymph nodes are slightly edematous, swollen and lightly stained, and the arrangement of cells in the germinal center is slightly loose and disordered (black arrow)	Some cells in the germinal center of lymph nodes were mildly edematous, with swollen cells and pale staining of cytoplasm, and the arrangement of cells in the germinal center was slightly loose and disordered (black arrow); Diffuse infiltration of inflammatory cells can be seen in the interstitial area of germinal center of tissue lymph nodes (red arrow)	Some cells in the germinal center of lymph nodes were moderately edematous, with swelling of cells and pale staining of cytoplasm, and the arrangement of cells in the germinal center was slightly loose and disordered (black arrow); A large number of inflammatory cells were diffusely infiltrated in the interstitial area (red arrow)	Some cells in the germinal center of lymph nodes were severely edematous, with swelling of cells and pale staining of cytoplasm, and the arrangement of cells in the germinal center was slightly loose and disordered (black arrow); A large number of coagulative necrosis of germinal center cells and severe structural damage (green arrow); A large number of inflammatory cells were diffusely infiltrated in the interstitial area (red arrow)	A large number of coagulative necrosis of germinal center cells, severe destruction of germinal center structure, and disappearance of boundary (green arrow); Massive red blood cell aggregation (blue arrow) due to local bleeding of tissue
**Representative pathological presentation**	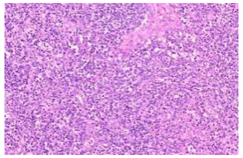	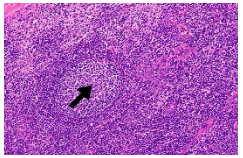	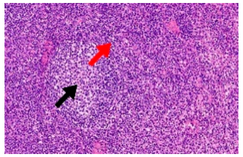	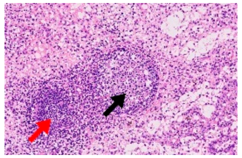	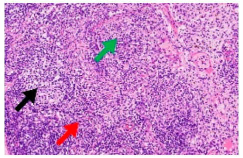	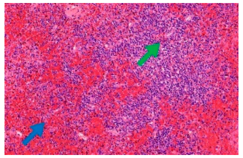

**Organic tissue**	**Mesenteric Lymph Nodes**					
**Lesion score**	0	1	2	3	4	5
**Lesion description**	Health	Some cells in the germinal center of lymph nodes were slightly edematous, and the arrangement of cells in the germinal center was slightly loose and disordered (black arrow)	Some cells in the germinal center of lymph nodes were slightly edematous, and the arrangement of cells in the germinal center was slightly loose and disordered (black arrow); Diffuse infiltration of inflammatory cells can be seen in the interstitial area of germinal center of tissue lymph nodes (red arrow)	Some cells in germinal centers of lymph nodes were moderately edematous, swollen and lightly stained, and some cells in germinal centers of lymph nodes were disorderly arranged and moderately scattered in morphology and structure (black arrow); Diffuse infiltration of inflammatory cells can be seen in the interstitial area of germinal center of tissue lymph nodes (red arrow)	Some cells in the germinal center of lymph nodes were mildly edematous, with swollen cells and pale staining of cytoplasm, and the arrangement of cells in the germinal center was slightly loose and disordered (black arrow); Diffuse infiltration of inflammatory cells can be seen in the interstitial area of germinal center of tissue lymph nodes (red arrow)	A large number of cells in the germinal center of lymphoid tissue are pyknotic and necrotic, and the germinal center structure is seriously scattered, and the tissue morphological structure is damaged (black arrow)
**Representative pathological presentation**	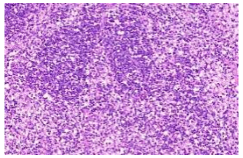	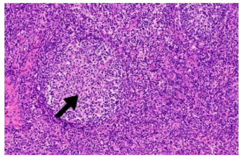	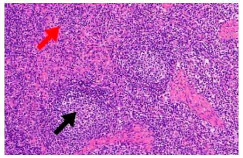	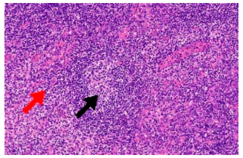	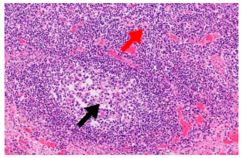	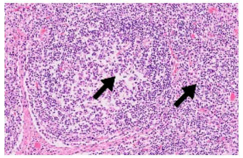
